# Financial assistance programs for cancer patients

**DOI:** 10.25082/ccr.2021.01.007

**Published:** 2021-09-17

**Authors:** Steven S. Coughlin, Lorraine T. Dean, Jorge E. Cortes

**Affiliations:** 1 Department of Population Health Sciences, Medical College of Georgia, Augusta University, Augusta, GA, USA; 2 Institute of Public and Preventive Health, Augusta University, Augusta, GA, USA; 3 Department of Epidemiology, Johns Hopkins Bloomberg School of Public Health, Baltimore, MD, USA; 4 Department of Oncology, Johns Hopkins Bloomberg School of Medicine, Baltimore, MD, USA; 5 Department of Medicine, Medical College of Georgia, Augusta University, Augusta, GA, USA; 6 Georgia Cancer Center, Augusta University, Augusta, GA, USA

**Keywords:** cancer, costs, financial distress, financial toxicity

## Abstract

**Background:**

The high costs of oncology care can lead to financial stress and have deleterious effects on the well-being of patients and their families. However, only a handful of financial assistance programs for cancer patients have been implemented and evaluated to date.

**Recent findings:**

Key features of reported programs include instrumental support through financial navigation or education for patients, and financial or charitable support for healthcare costs. Only one of the programs successfully reduced actual out-of-pocket costs for patients, though others were associated with psychosocial benefits or increased knowledge of financial resources. Four of the 5 programs evaluated to date were pilot studies with small sample sizes, and most lack control groups for comparison.

**Conclusions:**

Additional studies are needed that include larger sample sizes and with comparison groups of cancer patients in order to determine whether the counseling and navigator programs are effective in addressing financial distress in this patient population. Of particular interest are programs designed for low-income patients and those who lack health care insurance. Financial assistance programs that implement solutions at different levels of the healthcare system (individual patients, providers, healthcare institutions) are more likely to be effective. Multi-level interventions are needed that address the systems in which patients access care, the actual costs of services and drugs, and the individual needs of patients in order to reduce financial hardship for cancer patients.

## 1 Background

In the past two decades, a burgeoning literature has emerged documenting the problem of financial distress among cancer patients [1-4]. The high costs of oncology care can lead to financial stress and have deleterious effects on the well-being of patients and their families [5]. Financial hardship in cancer patients has been shown to be associated with decreased treatment adherence, poorer quality of life, and worse survival [6]. Cancer patients may struggle to pay out-of-pocket expenses due to the high expenses incurred, the medical debt, or loss of work [2, 7]. Women, younger patients, racial and ethnic minorities, low-income patients, and those without health insurance have an increased risk of financial distress [8, 9]. A framework of financial hardships developed by Altice *et al*. [10] considers three areas of hardship: material conditions (actual costs to the patient, including direct and indirect costs), psychological response (stress caused by the direct/indirect costs of care), and coping behaviors (behavioral responses like skipping medications or delaying care). This framework has been widely adopted within oncology.

While the high prevalence and importance of financial distress among cancer patients is clear from published studies, only a handful of financial assistance programs for cancer patients have been implemented, evaluated, and reported to date.

## 2 Methods

The present review is based upon bibliographic searches in PubMed and CINAHL and relevant search terms. Articles published in English through December 1, 2020 were identified using the following MeSH search terms and Boolean algebra commands: (financial distress OR financial toxicity) AND cancer. The searches were not limited to words appearing in the title of an article nor to studies in a particular country or geographic region of the world. The references of review articles were also reviewed. Information obtained from bibliographic searches (title and topic of article, information in abstract, study design, and keywords) was used to determine whether to retain each article identified in this way. Only intervention studies written in English that examined financial distress/toxicity in cancer patients were eligible for inclusion. A total of 5 articles were identified in the bibliographic searches that met the study criteria

## 3 Results

Shankaran *et al*. [6] developed a financial navigation program in collaboration with Consumer Education and Training Services (CENTS) and the Patient Advocate Foundation (PAF), to improve patient knowledge about treatment costs, provide financial counseling, and to help manage out-of-pocket expenses (Table 1). The PAF is a national organization that assists patients with a range of issues, including access to health insurance coverage, drug copayment assistance, coverage denials, transportation and meals, debt relief, and disability applications [6]. In a pilot study, 34 cancer patients with nonmetastatic solid tumors received an in-person or online financial education course followed by monthly contact with a financial counselor and a PAF case manager for 6 months. Caregivers were encouraged to participate in all aspects of the program. The educational content focused on money management, finding copayment assistance for high-cost drugs, and understanding and navigating health insurance plans. The financial counselors assisted with budgeting, retirement planning, and questions about medical bills. A counselor assigned to each patient arranged an initial meeting to review the patient’s expenses, income, and assets, and to provide a prospective budget or financial strategy. The case managers assisted with applications for insurance coverage, cost of living issues (for example, transportation), and disability applications. High financial burden and anxiety about costs were reported at baseline by 37% and 47% of patients, respectively [6]. Although self-reported financial burden did not change over time, anxiety about costs decreased in 33% of patients.

A follow-up to this pilot study [11] expanded to caregivers included a financial education video, monthly contact with a CENTS counselor and PAF case manager for 6 months, and referral to the non-profit charitable assistance program, Family Reach, for help with unpaid cost-of-living expenses (*e.g.*, transportation, housing). Outcomes were measured as baseline and follow-up reports of patient financial hardship using the Comprehensive Score for Financial Toxicity–Patient-Reported Outcomes [12] and caregiver work and financial strain using the Caregiver Strain Index (CSI) measure [13]. Among 32 patients and 18 primary caregivers for those patients, financial burden and caregiver stress did not change significantly during the pilot despite the fact that participants received mean of $772 per household (with an additional $647 for some families with non-medical expenses) which may not be enough to overcome stress due to the comparative thousands of dollars for the costs of cancer care. The authors note that the type of assistance needed varied by the participant’s income level, with lower-income individuals most often needing help to meet basic cost-of-living expenses such as transportation and housing (66% of participants with incomes of less than $50,000 received cost-of-living assistance), and higher-income participants needing help with issues of employment, medical cost coverage, and insurance navigation.

The PAF was also used in a single-arm pilot oncology navigation program for 12 brain cancer patients by Sadigh, *et al*. [14]. This study used the COST tool [12], as a measure of financial distress at study entry and 3, 6, and 9, and 12-month following and tracked the number of issues patients discussed regarding debt, disability, employment, insurance, medical decision-making and psychosocial support. At 3 months, five patients who completed a follow-up COST tool measurement showed no significant difference in scores from baseline; two of the total 12 patients completed the COST tool at 12 months, with only one-point difference from initial COST assessment. Patients discussed 12 total issues with PAF counselors, of which 93% were resolved within 6 months, with a total of $15,110 debt relief assistance provided.

In a pilot study conducted by Kircher *et al*. [16] 95 patients with advanced solid cancers receiving intravenous chemotherapy were randomly assigned to a financial counseling intervention or to standard care. The financial counseling intervention included a telephone and in-person consultation with a financial counselor that included health insurance education and an estimate of out-of-pocket expenses and total billed charges for one cycle of chemotherapy, including cost of drug, administration, and supportive intravenous medications. Seventy-six percent of patients reported having no difficulty understanding the information, suggesting high clarity. The results suggested that the telephone intervention was more feasible than the in-person consultation with a financial counselor. The authors noted that inclusion of responsible household members is important as patients may not handle their insurance or financial issues.

While most studies have focused on providing education or assistance to patients, one study focused on training staff on how to administer financial assistance. Yezefski *et al*. [15] trained financial navigators at 4 hospitals through the NaVectis Group, an organization that provides training to healthcare staff to increase patient access to care and assist with out-of-pocket expenses. Key components of the program included providing education and training to healthcare staff on how to improve patient access to financial assistance, implementing systematic processes for identifying patients in need, obtaining or improving insurance coverage for patients, and using tracking software to quantify benefits. To evaluate the impact of the program, data regarding financial assistance and hospital revenue were collected after instituting these programs. Outcomes for patients qualifying for assistance included: annual counts of the number of patients receiving navigation, the amount of assistance, and the types of assistance (free medication, new insurance enrollment and benefit maximization, premium/co-pay assistance, transportation, medical equipment). Outcomes were assessed from 2012-2016, with each hospital participating in the program for 1 up to 5 years (totaling 11 years of observation time), and offering different sets of services. Across all hospitals, an average of 32% (n = 3,572) of 11,186 new patients with cancer seen between 2012 and 2016 qualified for assistance. Trained financial navigators saved patients $39 million in financial assistance, an average of $3.5 million per observation year [15]. Patients saved an average of $33,265 annually on medication most often through connecting patients to foundations or pharmaceutical assistance programs, $12,256 through assistance with enrollment in insurance plans, $35,294 with premium assistance paid by the hospitals, $3076 through referrals to co-pay assistance programs. Referrals to community assistance programs helped patients with non-medical direct costs such as transportation, averaging $900 per patient. Hospitals benefited by avoiding write-offs and saving on charity care by an average of $2.1 million per year [15]. While the program was associated with cost-savings for patients and hospitals, the authors note that they did not have similar hospitals that did not use trained financial navigators to which to compare results.

## 4 Discussion

Key features of the reported programs included instrumental support through financial navigation or education for patients, and some provided financial or charitable support for healthcare costs. One program successfully reduced actual out-of-pocket costs for patients [15]. Although evaluative results from financial counselor and financial navigator interventions are promising [6, 15, 16], the number of programs is few. Four of the 5 programs evaluated to date were pilot studies with small sample sizes, and most lack control groups for comparison. Additional studies are needed that include larger sample sizes and with comparison groups of cancer patients in order to determine whether the counseling and navigator programs are effective in addressing financial distress in this patient population. Of particular interest are programs designed for low-income patients and those who lack health care insurance.

As institutions consider developing new interventions, educational components should focus on: educating patients about the basics of health insurance and costs they may face during treatment, as well as patient financial assistance programs; making patients aware of potential costs by providing estimates of cancer care costs; and offering education and assistance to patients about insurance benefits and other financial aid that may be available to them [17]. Nonetheless, the lack of cost-savings for education interventions suggest that cost transparency or education on costs will not be enough to sufficiently reduce patient cost burden.

While education-based interventions may help patients to manage anxiety around financial burden, increasing awareness of costs does not decrease costs, and does not solve the underlying issue that costs are too high. Thus, in addition to educational programs, the incentive systems that keep costs high need to be changed. The results of the existing interventions suggest that lowering the costs of care, offering instrumental support through navigation services that connect patients directly to assistance, or having hospitals cover the cost of care when needed are more successful strategies for reducing costs than awareness.

Other forms of instrumental support may include connecting patients to other types of assistance programs. Prescription assistance programs are offered by pharmaceutical manufacturers and charitable foundations to provide medications at reduced or no cost to medically indigent patients [18]. However, identifying strategies to assist with medications may be valuable but may not reduce overall burden or anxiety about cancer-related financial hardship. Nevertheless, prescription assistance programs provide a valuable safety net to help ensure that uninsured cancer patients receive needed prescription medications [18]. Manufacturer-sponsored copay assistance programs generally provide support for a specific drug but unfortunately Medicare beneficiaries are not typically eligible for these programs [19]. However, both patients with private insurance and Medicare beneficiaries can receive support from charitable co-payment assistance programs [19]. There is however little information on the reach of these programs, including the percentage of those helped among applicants, the effect on adherence and outcome and other elements that would be valuable to better understand their reach and limitations.

Financial assistance programs that implement solutions at different levels of the healthcare system (individual patients, providers, healthcare institutions) are more likely to be effective [5]. For example, at the hospital level, strong measures that hospitals can take to address financial toxicity include cost transparency and provision of financial counselors [5]. Interventions addressing laws governing drug pricing, provider reimbursement, and addressing the role of pharmacy benefit management on insurance premiums [20] can influence cost-savings for both care and medications. We need multi-level interventions that address the systems in which patients access care, the actual costs of services and drugs, and the individual needs of patients in order to truly reduce financial hardship for cancer patients.

## Figures and Tables

**Table 1:** Studies of financial assistance programs for cancer patients

Author (location)	Design	Outcomes	Sample	Results
Shankaran et al. [6] (Seattle, WA)	Non-randomized pilot study of an in-person or online financial education course followed by monthly contact with a financial counselor and a case manager for 6 months.	Self-reported financial burden and anxiety about costs	34 cancer patients with nonmetastatic solid tumors	High financial burden and anxiety about costs were reported at baseline by 37% and 47% of patients, respectively [6].
Watabayashi et al. [11] (Seattle, WA)	Non-randomized pilot study	Baseline and 6-month follow-up reports of patient financial hardship using the Comprehensive Score for Financial Toxicity–Patient-Reported Outcomes and caregiver work and financial strain using the Caregiver Strain Index measure	32 cancer patients and 18 primary caregivers for those patients	Financial burden and caregiver stress did not change significantly during the pilot despite the fact that participants received a mean of $772 per household (with an additional $647 for some families with non-medical expenses).
Sadigh et al. [14] (Atlanta, GA)	Non-randomized pilot study	This study used the COST tool as a measure of financial distress at study entry and 3, 6, and 9, and 12-months and tracked the number of issues patients discussed regarding debt, disability, employment, insurance, medical decision-making and psychosocial support.	12 brain cancer patients	At 3 months, five patients who completed a follow-up COST tool measurement showed no significant difference in scores from baseline; two of the total 12 patients completed the COST tool at 12 months, with only one-point difference from initial COST assessment. Patients discussed 12 total issues with counselors, of which 93% were resolved within 6 months, with a total of $15,110 debt relief assistance provided.
Kircher et al. [16] (Chicago, IL)	Two-arm randomized controlled trial	Self-reported financial distress, health-related quality of life, and acceptability	95 patients with advanced solid cancers receiving intravenous chemotherapy	No significant changes in financial distress were found between arms. Seventy-six percent of patients reported having no difficulty understanding the information, suggesting high clarity.
Yezefski et al. [15]. (four hospitals in the United States)	Non-randomized evaluation study	Data on financial assistance and hospital revenue. Outcomes for patients included: annual counts of the number of patients receiving navigation, the amount of assistance, and the types of assistance (free medication, new insurance enrollment and benefit maximization, premium/co-pay assistance, transportation, medical equipment).	Financial navigators at 4 hospitals	Trained financial navigators saved patients $39 million in financial assistance, an average of $3.5 million per observation year [15]. Patients saved an average of $33,265 annually on medication most often through connecting patients to foundations or pharmaceutical assistance programs, $12,256 through assistance with enrollment in insurance plans, $35,294 with premium assistance paid by the hospitals, $3076 through referrals to co-pay assistance programs.
